# Biomedical Approaches to HIV Prevention

**Published:** 2010

**Authors:** Kenneth H. Mayer, Margie Skeer, Matthew J. Mimiaga

**Keywords:** Alcohol and other drug use, abuse, and dependence, human immunodeficiency virus (HIV), HIV prevention, risk factors, sexual behavior, treatment

## Abstract

People who use and abuse alcohol and other drugs are an important population to target for HIV prevention because they are more likely to engage in sexual behaviors that increase their likelihood of acquiring or transmitting HIV. A variety of biomedical approaches to HIV prevention have been evaluated or currently are being studied. These approaches include an anti-HIV vaccine; topical protection treatments; and additional biomedical and barrier approaches, such as controlling sexually transmitted diseases, male circumcision, diaphragm use, and substance abuse treatment. The article also reviews topical versus oral antiretrovirals to prevent HIV transmission, antiretroviral treatment as prevention, and the role of alcohol and other drug use in HIV prevention.

Approximately 80 percent of the more than 60 million people infected with the human immunodeficiency virus (HIV) since the epidemic was first detected more than 25 years ago were infected via sexual intercourse (The United Nations Joint Programme on HIV/AIDS [UNAIDS]). Ironically, this mode of transmission is not particularly efficient. Early studies estimated the rate of HIV sexual transmission to be less than 1 per 2,000 for coital acts with an infected partner (Royce et al. 1997). Certain sexual practices are more efficient in facilitating HIV transmission than others. For example, receptive anal intercourse may have an efficiency as high as 1 per 10, although other studies have found it to be as inefficient as 1 per 6,000 (Shattock and Moore 2003). The high variability in the estimates of the efficiency of HIV transmission stems from two factors. First, because it would be unethical to observe HIV transmission in real time, studies of transmission are based on interviews with newly infected people. These studies may collect data over several months, during which multiple risky exposures could occur. Second, there is substantial biological variability in factors that facilitate infectiousness and susceptibility. For example, anything that increases the level of HIV concentration in blood will be associated with the efficiency of sexual transmission. Therefore, people who are acutely infected with HIV, who experience increased plasma viremia, or those who have advanced HIV disease would be more likely to transmit to their partners (Wawer et al. 2005). Other factors associated with increased transmission rates include concomitant and genital tract infections; other causes of local inflammation, stage of infection (acute, early, latent, or late); concurrent sexually transmitted diseases (STD) and coinfections; vaccines, which can increase viral load; and pregnancy (Chan and Ray 2007; Cohn 2004; Gray et al. 2005; Modjarrad et al. 2008; Ostrowski et al. 1997). The most prominent factor associated with decreased likelihood of becoming infected is male circumcision because the male foreskin contains many cells that can bind HIV (Bailey et al. 2007; Gray et al. 2007). Genetic factors which may alter the cellular receptors which can bind HIV, rendering some hosts less susceptible to HIV, and other genetic loci may make some people more capable of mounting an effective response to initial HIV infection (Rowland-Jones et al. 2001).

It also is feasible that medication to inhibit HIV (i.e., antiretroviral therapy) may decrease the likelihood of an infected person transmitting HIV to his or her partner (Cohen et al. 2002). Unfortunately, despite the increased access to antiretroviral therapy, there are more than 2.5 million new HIV infections occurring across the globe (UNAIDS 2007). Some feel that increased access to antiretroviral therapy (Granich et al. 2009), or even providing antiretroviral therapy only to symptomatic individuals (Wagner and Blower 2009), might be able to drastically curb the rates of new infections. Others note, however, that before antiretroviral therapy can have an appreciable impact on the epidemic, all patients taking the medication would need to be fully adherent and have suppressed viral loads all the time; have optimal management for genital-tract inflammation, like sexually transmitted infections (STIs); and have to not increase risk-taking behavior on the belief that they may be less infectious. Thus, with the increased appreciation that lowering plasma HIV levels may make individuals less infectious, the translation to public health practice still is a work in progress. Programs designed to promote adherence and safer sex among substance users with HIV will continue to be extremely important.

Substance use is a common denominator that influences the effectiveness of HIV transmission prevention efforts. Alcohol consumption in particular has been associated with decreased inhibition and impaired judgment, and, as a result, an increased likelihood of engaging in unprotected intercourse (Bryant 2006; Colfax et al. 2004; Samet et al. 2007; Stueve and O’Donnell 2005). Furthermore, as medication is increasingly being used as a part of HIV prevention interventions (e.g., pre-exposure prophylaxis), the role of alcohol in decreased adherence will be an essential consideration. It is therefore important to understand the current status of biomedical interventions for HIV prevention and, in some cases, the ways in which alcohol can impede these efforts. This article reviews research to develop methods for preventing HIV transmission, including an anti-HIV vaccine, topical protection treatments, and additional biomedical and barrier approaches such as controlling STDs, male circumcision, diaphragm use, and substance abuse treatment. The article also will discuss topical versus oral antiretrovirals to prevent HIV transmission, antiretroviral treatment as prevention, and the role of alcohol and other drug use in HIV prevention.

## The Search for an Anti-HIV Vaccine

HIV resides in cells that are present in the genital tract and rectal tissues of HIV-infected men and women, and secretions from these cells contain both cell-free viral particles (i.e., virions) and cell-associated HIV (Anderson et al. 1991; Fideli et al. 2001; Quinn et al. 2000). Epidemiologic data, as well as data from animals and in vitro infections, suggest that either of these forms can be infectious (Anderson et al. 1991; Fideli et al. 2001; Quinn et al. 2000). Adaptive host immune response^1^ usually is composed of two branches. The humoral branch controls infections through the production of antibodies, which are proteins that are able to attach to a foreign substance, known as an antigen (e.g., a virus). Once antibodies are bound to an antigen, the invading pathogen can be destroyed through a number of intracellular and extracellular mechanisms. Production of highly active antibodies that can neutralize a variety of viral strains can result in complete protection against certain types of infections. For example, the production of hepatitis B surface antibody, either through natural infection or via vaccination, protects the individual from liver damage and reinfection with hepatitis B. Antibodies may be protective through other mechanisms as well, such as promoting interactions with the complement system. Thus far, vaccine-generated antibodies have not been shown to be sufficient in protecting individuals against HIV.

The second branch of the immune response to viruses involves the cellular immune response, which is a more complex series of responses that creates cells that recognize cells containing intracellular pathogens. Cells that are primed to produce a host defense response are known as cytotoxic T-lymphocytes. These cells, when activated against a specific antigen, can break down cells that contain foreign pathogens. When people first become HIV-infected, they make antibodies with some neutralizing abilities (Kwong et al. 1998). Rapid viral evolution, however, changes the proteins on the outer envelope of the virus, and this makes it difficult for the antibodies to recognize these new isolates (Wei et al. 2003).

Because HIV can be transmitted in animal models by both cell-free and cell-associated viral particles, it is likely that optimal prevention of HIV transmission will necessitate generating effective antibodies as well as cytotoxic T-lymphocytes. A variety of approaches have been used in human clinical trials, but none have yet been sufficiently able to produce an immune response (i.e., to be sufficiently immunogenic) to warrant licensure.

### Vaccine Studies

The largest studies of a subunit vaccine that was designed to produce antibodies were the VaxGen trials (Flynn 2005). These studies used antigens that mimicked the GP120 outer envelope subunit of HIV in an effort to produce broadly neutralizing antibodies. The trials, unfortunately, did not demonstrate protection with this approach. The most advanced study of a vaccine that was designed to produce cytotoxic T-lymphocytes, known as the Step Study (Buchbinder et al. 2008), did not result in protection against HIV seroconversion (i.e., replication of the virus to the point that a detectable level of antibodies has developed). In point of fact, this adenovirus vector vaccine might actually have increased susceptibility to HIV among men who were uncircumcised. These findings have raised many questions (Sekaly 2008). It is possible that the vaccine might have led to immunostimulation, which actually increased the number of potential HIV targets in the immunologically active foreskin. Results from a recent vaccine trial in Thailand demonstrated preliminary efficacy of a combination ALVAC–HIV and AIDSVAX B/E vaccine regimen among a community-based sample consisting mostly of relatively low-risk heterosexuals (Rerks-Ngarm et al. 2009). Although the results reached statistical significance, the effect sizes were quite modest and the mechanism of action of the combination vaccine regimen was unclear. Although the level of protection was not of sufficient magnitude for promotion as a public health prevention strategy, the results of this trial may be useful in guiding future HIV vaccine research and suggest that combination vaccination strategies may be efficacious. It may be possible to enhance the immunogenicity of adenovirus vaccines by boosting them with naked DNA containing antigens similar to those delivered by the viral vector. This approach currently is being studied in HVTN 505, which is a proof-of-concept study designed to determine if using a vaccine that presents more antigens to at-risk persons produces a broader immune response that may be at least partially protective.

As a result of the findings from the VaxGen and Step studies (i.e., that the vaccines did not provide HIV protection), many HIV vaccine researchers have retreated to more basic discovery issues. Researchers are using animal models, particularly those involving nonhuman primates, to study different delivery systems in order to enhance immunogenicity (Rao et al. 2006). Although the initial vaccine trials were looking for vaccines that could completely prevent HIV infection, the increasing recognition of the complexity of human immune systems, and the ability of HIV to evade immune responses, has resulted in newer vaccine trials attempting to look at correlates of protection (i.e., the immune responses mounted by people who seem to have the best protection from the specific vaccine). In addition, some of the newer vaccine trials are considering whether people who become infected after receiving the vaccine are better able to control HIV infection. These studies are based on the idea that if a vaccine cannot be fully protective against acquiring HIV, it may help a person who becomes infected to better control viral replication for many years, thus delaying the need to initiate antiretroviral therapy. This would allow the person to stay healthier for a longer period of time and may make the people who receive the vaccine less infectious to future partners. Although there have been many new and exciting developments in immunology over the past few years, it is now unlikely that a safe and effective HIV vaccine will be available within the next 5 years.

## Topical Protection

Topical microbicides are products that may be formulated as gels, sponges, films, or rings that can be applied to vaginal or rectal mucosa with the goal of preventing or significantly reducing the risk of acquiring STIs, including HIV (Stein 1990). One of the first products studied as a topical microbicide, the spermicidal agent Nonoxynol-9 (N-9), unfortunately was shown to be neither safe nor effective (Hillier et al. 2005; Van Damme et al. 2002). There currently are almost 80 candidate microbicides in development (McGowan 2006). However, most of these candidates have been studied either in test tubes or in animal studies, and relatively few human studies have been fully evaluated. The current group of microbicides act through several different mechanisms of action, including (1) vaginal defense enhancers that help maintain the acidic vaginal pH, which is protective against foreign microbes. This has been studied as both a gel that can be applied topically and through the development of efficient recolonizing bioengineered lactobacilli, because they produce the majority of the hydrogen peroxide that are ultimately responsible for the acidic vaginal environment. (2) detergents that disrupt microbial membranes. (3) entry or fusion inhibitors that target viral or cell receptors to prevent the sequence of viral binding, fusion, and entry; and (4) inhibitors of viral replication. Some topical microbicides that were evaluated in clinical trials, cellular sulfate and C31g (Savvyä), were shown to be ineffective in protecting against HIV transmission (Van Damme et al. 2002). However, the most recent large-scale efficacy trial comparing PRO2000, a microbicide to inhibit HIV entry, with an acidifying agent, Buffergel, suggested that those receiving PRO2000 had a 30 percent decreased risk of HIV transmission compared with a nongel comparison group and a placebo control group (Abdool-Karim et al. 2009). The women who were most adherent to using the PRO2000 gel, particularly those who engaged in unprotected intercourse, seemed to have even higher levels of protection. Because 30 percent efficacy is at a borderline level for public health significance, this first positive finding in a microbicide efficacy trial will not lead to immediate licensure of PRO2000. A second, larger study conducted by the Medical Research Council of the United Kingdom is underway in East Africa. If that study shows that PRO2000 is at least as effective as the levels of protection seen in the earlier study, HVTN 035, then it is very likely that PRO2000 could be the first licensed topical microbicide agent.

Although there are microbicides being studied with varying mechanisms of reactions, the largest growth in microbicide research in recent years has been in the studies of the use of topical antiretrovirals for microbicidal protection. The first study of a topical antiretroviral compound evaluated topical tenofovir gel (Mayer et al. 2006). This study found that the gel was safe and well tolerated and, in a small subset of HIV-infected women, did not rapidly lead to the development of resistant strains. In addition, for about half of the women who participated in a pharmacokinetic substudy, detectable of levels of tenofovir were found in the blood that would be much lower than those used to treat HIV infections. These findings raise both concerns and hopes. The ability to absorb an antiretroviral drug topically delivered to the mucus membranes of the vagina could select for resistant variants because of the exposure of the viral particles in the semen of an infected partner to subinhibitory concentrations of medication. However, these results also might suggest that very high drug levels are being distributed to the genital tract submucosa, where the virus might normally try to replicate once it had infected superficial epithelial cells. Thus, the promise and concern about topical antiretrovirals will need to be carefully monitored in future clinical trials. Studies of topical microbicides have increasingly overlapped with studies of the use of systemic chemoprophylaxis (i.e., pre- or postexposure prophylaxis) to prevent HIV transmission (see below).

## Additional Biomedical and Barrier Approaches

### STD Control

A number of intervention trials have been conducted to test the efficacy of improving the management of STDs as an HIV-prevention strategy. The results are mixed, however, depending on the stage of the epidemic. One early trial in Mwanza (Grosskurth et al. 1995) that trained healthcare providers to treat STDs and provided medication for STDs was shown to be efficacious in preventing HIV. However, subsequent trials have not found generalizable principles to demonstrate this strategy as effective. A study in Rakai, Uganda (Wawer et al. 1999), utilized home-based antibiotic treatment to prevent the spread of HIV and concluded that HIV acquisition was independent of treatable STDs. It is possible that prevention efforts that focus on STD control may work better during earlier stages of the epidemic, as was the case in Mwanza, but not Rakai, and may need to address a complex array of STDs, ranging from bacterial infections, like syphilis and gonorrhea, to chronic viral infections, like *Herpes simplex*.

### Male Circumcision

Three randomized controlled trials have examined the utility of male circumcision as an HIV-prevention strategy among heterosexual men. These studies (Auvert et al. 2005; Bailey et al. 2007; Gray et al. 2007) all found that male circumcision significantly reduced the risk of HIV acquisition by approximately 50 percent among uninfected men. However, this approach may not be effective for other groups. For example among women, one study showed that HIV-infected men who were circumcised did not become less infectious to their female partners, which may be a result of increased sexual activity by the men too soon after the procedure (Wawer et al. 2008). The effects of male circumcision on men who have sex with men (MSM) have not been studied in clinical trials and would, at best, be protective only for men who did not engage in receptive anal intercourse.

### Use of Diaphragms

The Methods for Improving Reproductive Health in Africa (MIRA) trial examined the effectiveness of using a diaphragm with lubricant to prevent the acquisition of HIV among women in Zimbabwe and South Africa (Padian et al. 2007). The trial found that the use of diaphragms and lubrication, over and above the provision of condoms, did not afford woman added protection from HIV acquisition. Over the study period, the annual incidence among women who received diaphragms, lubricant, and condoms was 4.1 percent, whereas the annual incidence among women who only received condoms was 3.9 percent. Although the results indicated that the addition of diaphragms and lubricant was not better than condom use alone, the study was unable to assess whether the use of diaphragms and lubricant was more effective at preventing HIV infection than not using anything.

### Substance Abuse Treatment

Substance abuse treatment is an important HIV-prevention strategy because people in treatment are less likely to engage in risky sexual behaviors (Needle et al. 1998) and inject drugs or share needles (Fuller et al. 2009). Substance abuse interventions that impact HIV prevention in the U.S. include pharmacotherapy (e.g., opioid substitution; although no pharmacologic interventions have been shown to be effective for stimulant use) and behavioral interventions, including harm reduction techniques (e.g., needle exchange programs for injection drug users), as well as group and individual therapy.

## Topical versus Oral Antiretrovirals to Prevent HIV Transmission

Antiretrovirals may conceivably prevent HIV transmission by either reducing HIV concentrations in people who already are infected, or acting as pre-exposure prophylaxis (PrEP) or as postexposure prophylaxis (PEP). In the latter case, the compounds are being used to prevent HIV acquisition in HIV-uninfected people. As mentioned above, research is increasingly examining the desirability of applying topical gels versus using pills to prevent HIV transmission, given that many of the antiretrovirals in prevention studies are available as oral antiretroviral compounds.

Evidence supporting the use of PEP comes from the success of the prevention of mother-to-child HIV transmission (Conner et al. 1994), animal studies (Tsai and Follis 1995; Tsai et al. 1998), as well as a case– control study of prophylaxis after needle-stick injuries in health care settings (Cardo et al. 1997). Although no randomized controlled clinical trials have studied PEP, evidence from a retrospective case–control study of health care workers led to the adoption of PEP as standard of care in occupational studies (U.S. Public Health Service 2001). Following the recommendation for occupational PEP, several groups (Martin et al. 2004; Schechter et al. 2004) studied nonoccupational PEP (NPEP) and, in general, people who received NPEP were more likely to decrease their risk behavior over time, making it an “educable moment.” In addition, although there were failures of NPEP, there was very little transmission of antiretroviral-resistant strains. In the vast majority of cases, individuals were either nonadherent to the regimen and/or continued to engage in risky practices shortly after completing the antiretroviral treatment. In both occupational and nonoccupational PEP, treatment completion rates often are suboptimal because of side effects of many of the traditional three-drug regimens. An analysis by Bassett and colleagues (2004) found that because completion rates could increase with two-drug regimens, there might be a benefit to providing more simple regimens (Mayer et al. 2006). Other new approaches might use some of the better-tolerated drugs that inhibit the virus in unique ways (Mayer et al. 2009).

Animal studies also have suggested that protection may be conferred by antiretroviral drugs taken before a high-risk exposure (Subbarao et al. 2006). Studies have investigated different ways of challenging animals (i.e., monkeys) with different types of virus, but general themes have emerged suggesting that as long as antiretroviral drugs have achieved a sufficient concentration in the animal’s mucosa prior to the exposure, and as long as there is some postexposure drug dosing, the majority of the animals were protected (Garcia-Lerma et al. 2006). The failure of recent vaccine studies, nonspecific topical microbicides, the human diaphragm, and *Herpes simplex* suppression to prevent HIV transmission has led to increased interest in the use of antiretroviral PrEP. Concerns have been raised about possible behavioral inhibition among people in PrEP studies, as well as questions about the development of adverse reactions to the medication, acquisition of antiretroviral-resistant strains, and the mixed message of giving people pills and trying to counsel them to be safe. The first PrEP study that addressed these issues was conducted with 936 high-risk women in Ghana, Cameroon, and Nigeria and found that there were no significant adverse events in women who used oral tenofovir on a daily basis for chemoprophylaxis, and, moreover, the women’s risk behavior tended to decrease over time (Peterson 2007). Although the study was not sufficiently large to be able to show an efficacy effect, the fact that there were six infections among the women who received placebo and only two in the group that received tenofovir led to increased optimism about this approach for HIV prevention in high-risk populations. At the present time, efficacy trials are underway to study tenofovir with and without the drug emtricitabine (FTC) in MSM in the United States and throughout the world, as well as in high-risk heterosexuals in sub-Saharan Africa, HIV-discordant couples in sub-Saharan Africa, and drug users in Thailand. In addition, the HIV Prevention Trials Network (HPTN) is embarking on a study to test the feasibility of an enhanced “Test, Link to Care, Plus Treat” protocol (TLC-Plus) (HPTN 065, HPTN 2010). The first signals to suggest efficacy in these trials may be available in the next 1 to 3 years, but many questions will remain. One of the major questions is whether individuals can take fewer doses of medication in order to protect themselves from HIV acquisitions, or one dose before or after a high-risk exposure. Another question pertains to the potential for developing resistance once the drugs are more widely used for chemoprophylaxis. Although concerns have been raised about how these drugs may be widely used for prevention because of their availability from physicians who treat people with HIV, as well as their availability internationally in generic formulations, studies of high-risk populations have not found significant numbers of people already using chemoprophylaxis (Liu et al. 2006; Mimiaga et al. 2009). This is a dynamic area; as new data become available, it will be important to monitor the uptake of PrEP among high-risk populations and particularly those who are using alcohol and other substances that might put them at risk for decreased adherence and subsequent HIV infection.

## Treatment as Prevention

There are several lines of evidence to support the suggestion that antiretroviral treatment will reduce the infectiousness of treated patients, including retrospective analysis (Musicco et al. 1994), prospective observation studies, and ecological data (Castilla et al. 2005; Quinn et al. 2000). Several recent studies in Africa of HIV-discordant couples (Kayitenkore 2006) found that among 32 people who acquired HIV over a 3-year period, only 2 had an HIV-infected partner on antiretroviral therapy. The finding was replicated in a similar study of Ugandan patients initiating antiretroviral therapy; researchers reported a 98 percent reduction in the estimated risk of HIV transmission following the initiation of antiretroviral therapy (Bunnell et al. 2006). Although it has been suggested that wider-spread HIV testing and initiating antiretroviral therapy immediately in all patients could arrest the HIV epidemic (Granich et al. 2009), in settings where individuals are sexually active with multiple partners (e.g., MSM), medication adherence and safer sex practices become highly relevant issues. For example, despite the increasingly wide accessibility to antiretroviral therapy in San Francisco, the Department of Health there noted increases in incident HIV infection at the early part of this century, a finding that was replicated in Amsterdam (Dukers et al. 2002). To assess whether behavioral disinhibition and suboptimal medication adherence could play a role in decreasing the expected major benefit of antiretroviral treatment, the HPTN has a major study under way (HPTN 05TN) that will follow 1,750 HIV-discordant couples to assess whether early initiation of antiretroviral therapy has a beneficial effect in decreasing HIV transmission between partners.

## The Role of Alcohol and Other Drug Use in HIV Prevention

Alcohol use is associated with unprotected intercourse (Brown and Vanable 2007; Colfax et al. 2004; Graves and Hines 1997; Kalichman et al. 2007). However, some evidence from cross-sectional and event-level studies contradicts this association (Klitzman et al. 2000; Morrison et al. 2003; Springer et al. 2007). These discrepancies could be a result of the ways alcohol use was measured or variations in methods of data collection and analysis regarding the timing of sexual behavior and alcohol use (Drumright and Colfax 2009). Because alcohol consumption is linked to decreased inhibition and impaired judgment, and in light of contradicting data on the relationship between alcohol consumption and sexual risk behavior, experimental studies examining this phenomenon are warranted.

With increasing interest from the research community in the use of antiretroviral drugs for prevention, the role of behavioral scientists in these biomedical interventions is extremely important. People who have problems with alcohol, either through daily consumption or binge drinking, are more likely to be nonadherent and engage in HIV-transmitting behaviors (Cook et al. 2001; Samet et al. 2007; Silva et al. 2009). Thus, if the initial studies of PrEP, PEP, and the use of early-use antiretrovirals in HIV-infected patients prove to be successful in the controlled environment of a clinical trial, subsequent effectiveness studies will move to assess the impact of making these medications available to people with substance use problems. In addition, research will assess the need to develop tailored programs that will not only address the importance of taking the medications for prevention but also the reasons for substance use and try to develop effective interventions to minimize the impact of any substance use on medication adherence and risk-taking behaviors.

Because it is well documented that people who use and abuse alcohol and other drugs are more likely to engage in sexual behaviors that increase their likelihood of acquiring or transmitting HIV (Colfax et al. 2004; Samet et al. 2007; Stueve and O’Donnell 2005), targeting these people for PrEP will be an important step in curbing the incidence of HIV transmission. However, in addition to the issues related to substance abuse, a primary concern for this type of intervention would be the low rates of medication adherence inherent in substance-abusing populations. This is particularly pertinent, given that the use of alcohol and other drugs influences medication adherence among HIV-positive individuals, whose health depends on being highly adherent to antiretroviral regimens; when taking antiretroviral medication as a preventative strategy, levels of adherence may be even lower. For example, among HIV-infected individuals, those who believe it harmful to consume alcohol while taking antiretrovirals often stop their pill regimen while drinking (Kalichman et al. 2009). This practice may be even more prevalent among HIV-negative individuals, because of the elective nature of the regimen, especially if they feel that they must choose between taking their medication and drinking and/or using drugs.

A further concern for practitioners is the interaction between alcohol and other drugs with antiretroviral medication. Certain drugs, including amphetamines, benzodiazepines, and opioids, which are metabolized by the liver, may interact with antiretrovirals, making the combination potentially hazardous and therefore should be avoided (Antoniou and Tseng 2002). The effect of consuming alcohol while taking antiretrovirals has been shown to be less dangerous but may promote resistance and ultimately compromise the efficacy of the medications over time (Antoniou and Tseng 2002). These issues will have to be taken into consideration when deciding whether to offer individuals who use and abuse alcohol and other drugs PrEP for preventative purposes.

Myriad challenges arise when working with people who use and abuse alcohol and other drugs. Theses challenges notwithstanding, recommending PrEP as a preventative strategy with this population may prove to be both feasible and effective. If PrEP is found to be effective in preventing the transmission of HIV, it will be of great importance to identify and test the feasibility of this secondary prevention strategy among high-risk substance-using populations in order to curb rising rates of HIV among this at-risk group.

## Footnotes

1 For a definition of this and other technical terms, see the Glossary, pp. 288–291.
